# Optimization of dextran sulfate sodium-induced colitis and associated mortality in BALB/c mice

**DOI:** 10.1590/1414-431X2026e15450

**Published:** 2026-09-07

**Authors:** D.E. Molotla-Torres, M.E. Drago-Serrano, M. Godínez-Victoria, R. Oros-Pantoja, F. Guzmán-Mejía

**Affiliations:** 1 Ph.D. Program in Biological and Health Sciences, Metropolitan Autonomous University, Xochimilco Campus, Mexico City, Mexico; 2 Department of Biological Systems, Metropolitan Autonomous University, Xochimilco Campus, Mexico City, Mexico; 3 Graduate Studies and Research Section, Superior School of Medicine, Instituto Politécnico Nacional, Mexico City, Mexico; 4 Laboratory of Neuroimmunoendocrinology, Faculty of Medicine, Autonomous University of the State of Mexico, Toluca de Lerdo, Mexico

**Keywords:** Colitis, Dextran sulfate sodium, Intestinal permeability, Goblet cells, Mucopolysaccharides

## Abstract

Dextran sulfate sodium (DSS)-induced colitis in BALB/c mice has revealed underlying mechanisms of colitis pathology. This study aimed to optimize the DSS dosage to improve reproducibility while ensuring high survival rates. Mice were treated with 2 and 3% DSS solutions in drinking water daily for 7 days (days 1-7), followed by water (8-14 days). Disease activity index (DAI) and survival percentage were monitored over 14 consecutive days. On the last day, intestinal permeability was assessed by Evans blue assay, and colon length was measured. After sacrifice, colon samples were collected to evaluate mucopolysaccharide content and processed for histological analysis with hematoxylin-eosin (HE), periodic acid-Schiff (PAS), and Alcian blue (AB) staining. The results were analyzed to identify significant differences between groups, considering a P-value ≤0.05 as significant. Compared to the control group, DAI, inflammatory cell infiltrate, and intestinal permeability were higher, while colon length, body weight, and mucopolysaccharide content were lower in both DSS groups. Compared to the 2% DSS group, the 3% DSS group showed higher relative density of AB+ cells, higher DAI on days 7, 9, and 14, greater inflammatory infiltrate, and lower PAS+ cells. In addition, survival was lower in the 3% DSS group (57%) compared to the 2% DSS group (80%). According to these findings, 2% DSS provided higher survival and symptomatic colitis, making it the best choice for inducing DSS colitis in mice.

## Introduction

Ulcerative colitis (UC) is a chronic inflammatory disease localized in the colon and rectum. The underlying mechanisms leading to its pathogenesis have been explored in experimental models in mice, such as dextran sulfate sodium (DSS)-induced colitis, which primarily disrupts the gut epithelial barrier and subsequently triggers local inflammation (1). DSS is an acidic, negatively charged, sulfated polysaccharide readily soluble in water, with a molecular mass (MM) ranging from 5 to 1400 kDa. High MM DSS does not display toxic effects in the colon, whereas low MM DSS causes mild colitis. DSS with a 40-50 kDa MM has pronounced colitogenic activity when administered in drinking water to female BALB/c mice and reproduces features similar to human colitis (2). The preference of DSS for the colon as its target site appears to be related to the metabolic polarization of colonocytes, which may favor microbiota dysbiosis (3), and the high presence of medium-chain fatty acids (MCFA, 7-12 carbons) derived from dietary triglycerides (1).

DSS 40-50 kDa, along with MCFA, generates nano-vesicles that are prone to embed within membranes causing “blisters” that ultimately disrupt epithelial structural integrity, as described in female C57BL/6 mice (4). DSS-induced “blisters” on the colonic epithelial monolayer enable bacterial penetration into the inner mucus layer, facilitating contact with the apical membrane epithelium, as found in C57BL/c mice (5). Additionally, DSS-induced colitis severity may be associated with diarrhea, mucosal bleeding, and electrolyte loss (4,6,7). It has been suggested that the hemorrhagic effects of DSS may be related to its heparin-like properties (8). In DSS-induced colitis, increased translocation of luminal endotoxins into the blood circulation and an increased response of inflammatory cytokines such as tumor necrosis factor α (TNF-α) occur (9-12). DSS-induced colitis in BALB/c mice is a widely used model of experimental colitis (7,10-16). Assessing BALB/c mice survival to determine DSS severity and toxicity is critically important to establish the optimal DSS dosage that minimizes mortality and avoids unnecessary suffering, as required by ethical management of mice (10,17-20).

Despite the widespread use of DSS-induced colitis as an experimental model for ulcerative colitis, important limitations remain. Disease severity and mortality rates can vary substantially depending on mouse strain, DSS concentration, and exposure duration, often resulting in inconsistent experimental outcomes. In murine models, DSS administration can lead to variable susceptibility and increased mortality under certain experimental conditions, complicating the interpretation of inflammatory and histopathological parameters.

Therefore, the present study aimed to compare 2 and 3% DSS concentrations to optimize the induction of experimental colitis in BALB/c mice by evaluating mortality and selected gut barrier parameters. By identifying conditions that promote reproducible disease development while minimizing mortality, this study seeks to establish a more consistent and reliable protocol, thereby improving the utility of the DSS model for investigating mechanisms of intestinal inflammation and evaluating potential therapeutic interventions.

## Material and Methods

### Animals

Male BALB/c mice (22-25 g body weight) were provided by the Production and Experimentation Unit for Laboratory Animals (UPEAL), Universidad Autónoma Metropolitana Unidad Xochimilco (UAM-X). Before beginning the experimental interventions, mice were acclimated for one week in a room maintained at 22-24°C, 55% relative humidity, and a 12-h dark-light cycle (lights on at 7:00 AM and off at 7:00 PM), housed in groups of four per cage. Animals were fed a rodent diet and given purified water *ad libitum* (Laboratory Rodent Diet 5001; LabDiet, USA). Mice were handled according to the Manual of Organization and Procedures of the Internal Committee for the Care and Use of Laboratory Animals (CICUAL, protocol No. 231) of UAM-X, in compliance with Mexican Federal Regulations for Animal Experimentation and Care (NOM-062-ZOO-1999), Ministry of Agriculture, Mexico City, Mexico, and in accordance with the Animal Research Reporting of *In Vivo* Experiments (ARRIVE) guidelines (21). Male BALB/c mice were used to minimize hormonal variability and ensure reproducibility. This strain and sex combination provided a consistent response to DSS, making it suitable for modeling colitis severity and assessing intestinal damage.

### Experimental design

Animals were allocated into three groups - control, 2% DSS, and 3% DSS - with eight mice in each group. Three independent experimental assays were performed to evaluate different endpoints: intestinal permeability, mucopolysaccharide content, and histological analysis. This approach was necessary due to the limited tissue yield from the murine colon, which restricted the number of possible analyses. Survival, disease activity index (DAI), and colon length were assessed in all experiments; data for these parameters were pooled from the three independent experiments for analysis. The final number of animals analyzed for certain parameters was lower due to DSS-induced mortality.

### Experimental ulcerative colitis model

The experimental protocol for acute colitis was conducted using DSS Salt Colitis Grade (cat. No. 160110 MP Biomedicals LCC, USA) to prepare 2 and 3% weight/volume solutions dissolved in purified water.

Groups of eight mice were treated orally *ad libitum* with 2 or 3% DSS for seven consecutive days, followed by purified water from days 8 to 14. The control group received purified water throughout the experimental protocol.

### Survival monitoring and DAI

Mouse survival was monitored daily from the first day of the DSS protocol until sacrifice, and the survival rate is reported as a percentage (%) over 14 days. According to animal handling guidelines, severely ill mice were euthanized by overexposure to isoflurane.

The DAI was recorded daily for all surviving mice as described previously (22). DAI was evaluated according to the following parameters: 1) body weight, 2) stool consistency, and 3) fecal blood (hematochezia). The parameters were categorized according to the following score: “0” for no body weight loss, normal fecal consistency, and no hematochezia; “1” for 1-5% body weight loss, normal fecal consistency, and no hematochezia; “2” for 5-10% body weight loss, loose feces, and no hematochezia; “3” for 10-20% body weight loss, loose feces, and no hematochezia; and “4” for 20% or greater body weight loss, diarrhea, and hematochezia visible to the naked eye.

DAI was calculated as the sum of the clinical scores for body weight loss, stool consistency, and presence of fecal blood. Each parameter was scored on a scale from 0 to 4, resulting in a total DAI score ranging from 0 to 12. The area under the curve (AUC) of DAI was also recorded.

### Colon length and body weight

After completing the colitis protocol, surviving mice were euthanized by isoflurane overexposure. Following dissection, the full length of the colon was measured for each sample. Additionally, daily body weight (g) was recorded from days 1 to 14 as a parameter of colitis severity.

### Histopathology

After euthanasia, the colons were dissected and placed in jars containing 4% paraformaldehyde in phosphate-buffered saline (PBS, pH 6.9). The samples were stored for 48 h at room temperature (RT), washed twice with 70% ethanol, and embedded in paraffin. For histopathological analysis, tissues were stained with hematoxylin and eosin (HE). Periodic acid-Schiff (PAS) and Alcian blue (AB) staining were also performed to detect neutral and acidic mucins, respectively, according to previously described procedures with minor modifications (23). For histological analysis, samples were cut into 4-μm-thick sections, deparaffinized with xylene, and gradually rehydrated through a decreasing ethanol gradient.

For PAS staining, tissue sections were incubated in 0.5% PAS for 15 min. Afterward, samples were washed with distilled water, tap water, and PBS pH 7.4, incubated with Schiff's reagent for 20 min and then washed again as before. Subsequently, nuclei were counterstained with Harris hematoxylin for 3 min.

For AB staining, samples were washed with 3% glacial acetic acid for 3 min, stained with AB solution for 45 min, washed, and then transferred to 3% glacial acetic acid for 3 min. Subsequently, nuclei were stained with nuclear fast red solution for 10 min.

Cell morphology was visualized using light microscopy (Axiostar Plus, Carl Zeiss^®^, Jena, Germany) with Lumenera Infinity Analyze and Capture v. 6.5.4 software (Microsoft Windows 10, Lumenera Corp., Canada). To measure the size and number of goblet cells, images were analyzed using Image-Pro Plus v.7.0 software (Microsoft Windows 10, Media Cybernetics, Inc.).

### Evans blue permeability assay

On day 14 after completion of the colitis protocol, intestinal permeability was measured in surviving mice as previously described with some modifications (24). Briefly, 0.2 mL/20 g body weight of 0.25% w/v Evans blue dye dissolved in PBS pH 7.2 was intravenously injected into the caudal vein. One hour later, mice were sacrificed using isoflurane, and 3-cm colon strips were collected in pre-weighed 2-mL microcentrifuge tubes to determine the difference in sample weight. Colon samples were then mixed with 1 mL of formamide and incubated overnight at 50°C. Samples were centrifuged at 14,400 *g* for 5 min at RT, and the supernatant was collected in a clean 1.5-mL microcentrifuge tube to evaluate absorbance at 620 nm. The concentration of Evans blue in the colonic lumen was calculated based on a standard curve of Evans blue stock solution. Intestinal permeability is reported as Evans blue in μg/g tissue.

### Mucopolysaccharide quantification

After completing the colitis protocol, surviving mice were euthanized by isoflurane overexposure. The colon was dissected, and 1-cm colonic samples were inverted and weighed to quantify the total concentration of mucopolysaccharides using the AB assay based on the original method (25) with some modifications.

Colonic strips were immersed in 1.7 mL of 0.1% AB dye in 0.16 M sucrose dissolved in 0.05 M sodium acetate pH 5.8 and incubated for 2 h at RT under constant stirring. After incubation, samples were vortexed and centrifuged at 14,400 *g* for 10 min at RT. To remove excess supernatant from the pellet, two rounds of washing were i) pellet suspension in 1.7 mL of 0.25 M sucrose; ii) incubation at RT for 15 min under constant stirring; iii) vortex homogenization; iv) centrifugation at 14,400 *g* for 10 min at RT; and v) supernatant removal for pellet collection. The third and final round of washing was performed as before, incubating the pellet for 45 min in 0.25 M sucrose. To extract the dye bound to the mucus, the pellet was suspended in 1.7 mL of 30% docusate sodium dissolved in 70% ethanol and incubated overnight at RT under constant stirring. Finally, samples were thoroughly mixed and centrifuged at 14,400 *g* for 10 min at RT to obtain the supernatants, which were collected in clean 1.5-mL microtubes.

Serial dilutions of the supernatant from each sample were prepared in triplicate in a 96-well microtiter plate to a final volume of 100 μL in 30% docusate dissolved in 70% ethanol. Absorbance was read at a wavelength of λ=620 nm. Total mucopolysaccharide concentration was determined based on a standard curve prepared with different concentrations of chondroitin 4-sulfate (μg/μL) using 30% sodium docusate in 70% ethanol as the diluent, and the absorbance of each concentration was measured at 620 nm. Mucopolysaccharide concentration is reported as μg of AB per gram of tissue.

### Statistical analysis

Survival analysis was performed using the log-rank Mantel-Cox test. The results were subjected to the Kolmogorov-Smirnov normality test; data with a normal distribution were analyzed using one-way ANOVA and the Holm-Sidak *post hoc* test, while data with a non-normal distribution were analyzed using the nonparametric Kruskal-Wallis test and Dunn's multiple comparisons *post hoc* test. Differences with P≤0.05 were defined as significant. Data were analyzed using GraphPad Prism statistical software, version 8.0.1(USA).

## Results

### Survival percentage was greater with 2% DSS

**Figure 1:**
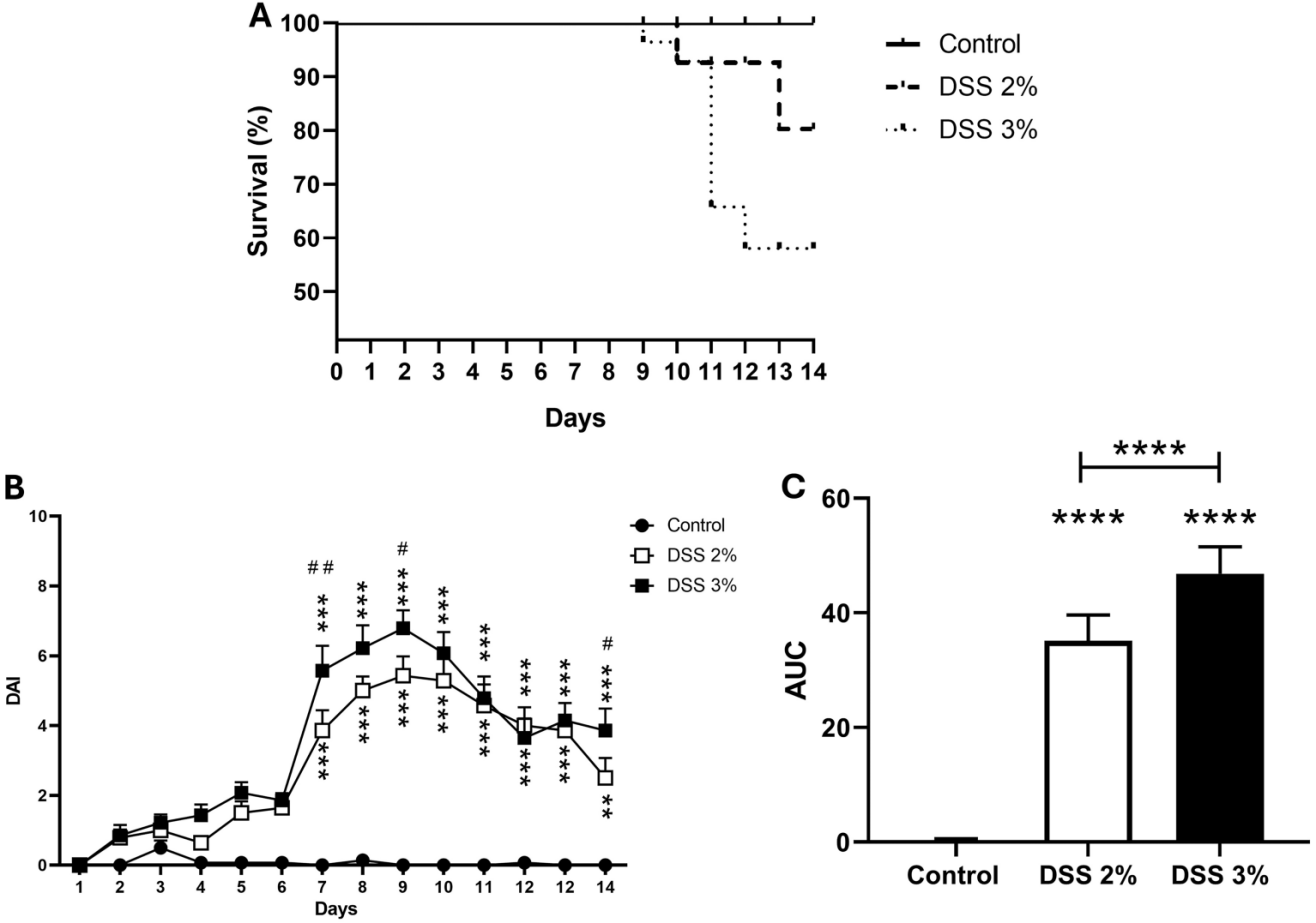
Progression and severity of colitis in BALB/c mice treated with 2 and 3% dextran sulfate sodium (DSS). **A**, Survival percentage. **B**, Disease activity index (DAI). **C**, Area under the curve (AUC) of data obtained from DAI values for each experimental group. Data are reported as means±SD of 24 mice per group. **P<0.01, ***P<0.001 *vs* control group; ^#^P<0.05, ^##^P<0.01 *vs* DSS-treated groups; ****P<0.0001 for AUC *vs* the control group and between DSS-treated groups. ANOVA and the Holm-Sidak *post hoc* test.

Survival was 100% in all groups from days 1 to 8. The 2% DSS group showed 90% survival from days 10 to 13 and 80% on day 14, whereas the 3% DSS group showed 95% survival on day 9, 90% on day 10, 62% on day 11, and 57% on days 12 to 14. Survival was higher in the 2% DSS group than in the 3% DSS group (P=0.015) (Figure 1A).

### DAI was greater in 3% DSS

DAI showed no significant differences among all groups from days 1 to 6 (Figure 1B). Compared to the control group, the DAI was higher in both DSS-treated groups from days 7 to 14 (P<0.001 for all days except P<0.01 on day 14 for the 2% DSS group). Comparisons between the DSS-treated groups indicated that DAI was significantly lower in the 2% DSS group than in the 3% DSS group on days 7 (P<0.01), 9, and 14 (both P<0.05). Compared to the control group, the area under the curve (AUC) for both DSS-treated groups was higher (P<0.0001). Furthermore, the AUC of the 3% DSS group was significantly higher than that of the 2% DSS group (P<0.0001) (Figure 1C).

### Colon length was decreased with both DSS concentrations, while body weight decreased only with 3% DSS

**Figure 2:**
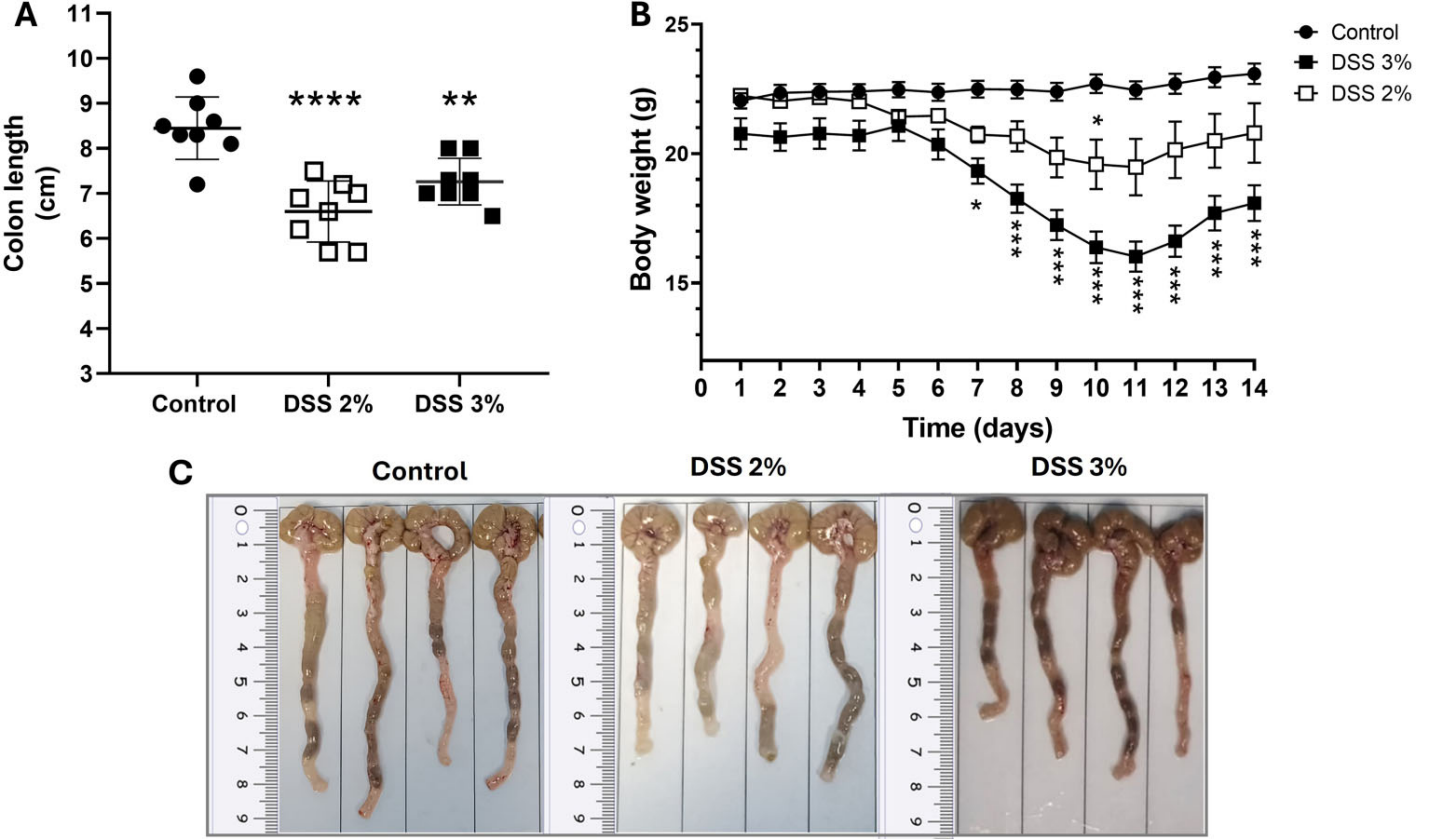
Colon length and body weight in dextran sulfate sodium (DSS)-induced colitis BALB/c mice treated with 2 and 3% DSS. **A**, Colon length (cm). **B**, Kinetics of body weight during the experimental period. **C,** Representative photographs of the colon for each group (scale=1 cm). Data are reported as means±SD of 8 mice per group. *P<0.05, **P<0.01, ***P<0.001, and ****P<0.0001 *vs* the control group. ANOVA and the Holm-Sidak *post hoc* test.

Compared to the control group, colon length was significantly shorter in both DSS-treated groups (P<0.0001 for 2% DSS and P<0.01 for 3% DSS), with no significant differences between them (Figure 2A and C).

Compared to the control group, body weight was significantly lower in the 3% DSS group on days 7-14, while in the 2% DSS group, it was only lower on day 10 (P<0.0001, except P<0.05 on day 7 for the 3% DSS group and day 10 for the 2% DSS group) (Figure 2B).

### Both dosages of DSS triggered tissue inflammation

**Figure 3:**
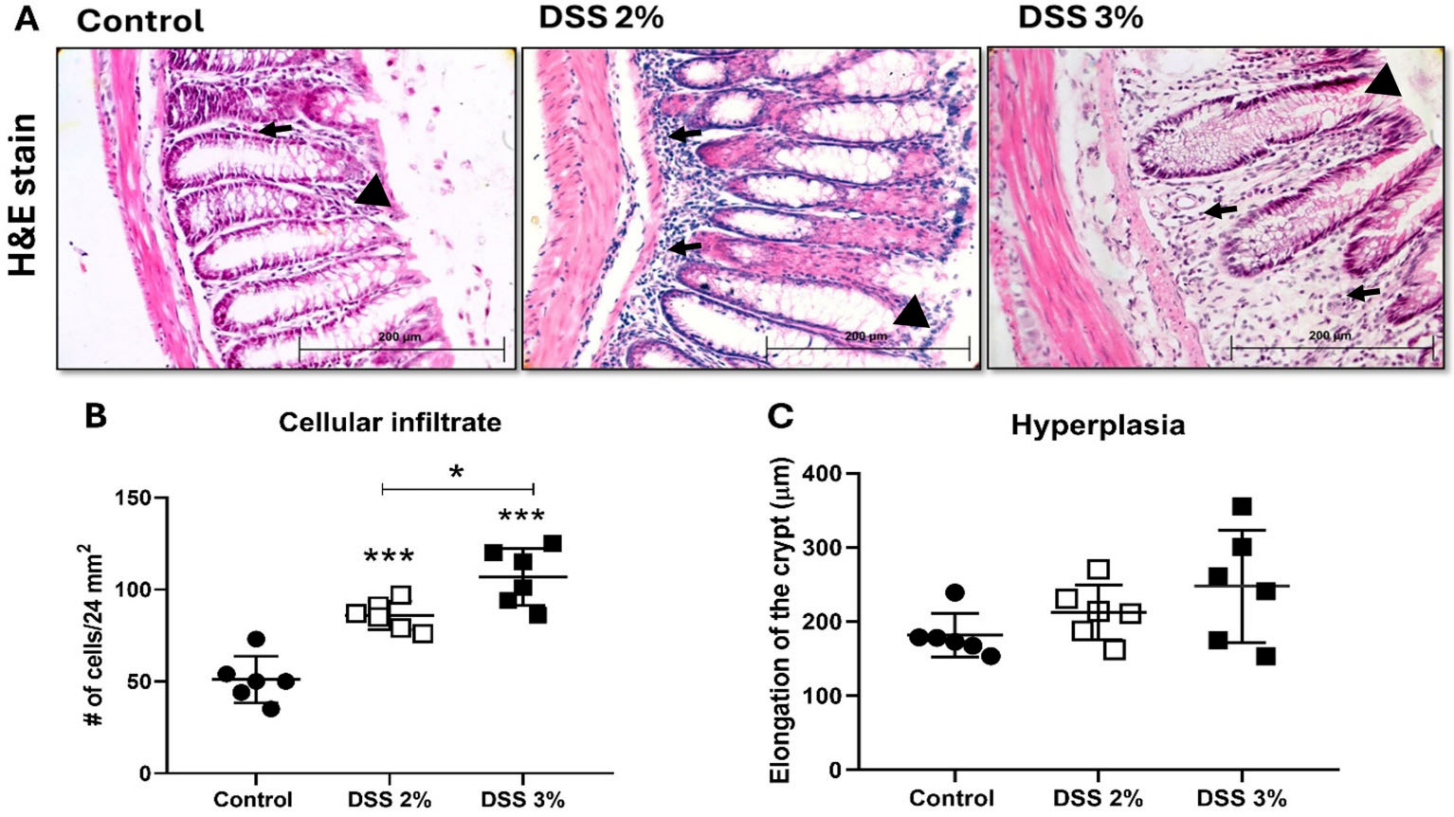
**A**, Hematoxylin eosin staining for tissue integrity analysis of colon samples of dextran sulfate sodium (DSS)-induced colitis in BALB/c mice treated with 2 and 3% DSS. Black arrows indicate cellular infiltrate in the mucosa and submucosa, while the black arrowheads show the crypt elongation (scale bar=200 μm). **B**, Cellular infiltrate computed by the number of cells per 24 mm^2^. **C**, Hyperplasia is reported as crypt elongation (μm). Data are reported as means±SD of 6 mice per group. ***P<0.001 *vs* the control group; *P<0.05 between DSS-treated groups. ANOVA and Holm-Sidak *post hoc* test.

HE staining for tissue integrity analysis (Figure 3A) showed that, compared to the control group, both DSS-treated groups exhibited loss of tissue integrity (P<0.001). Moreover, cellular infiltrate was lower in the 2% DSS group than in the 3% DSS group (P<0.05) (Figure 3B). Compared to the control group, both DSS groups showed an apparent increase in hyperplasia, but no significant differences were found between the DSS-treated groups (Figure 3C).

### DSS decreased acidic (2%) and neutral (3%) sugar levels

**Figure 4:**
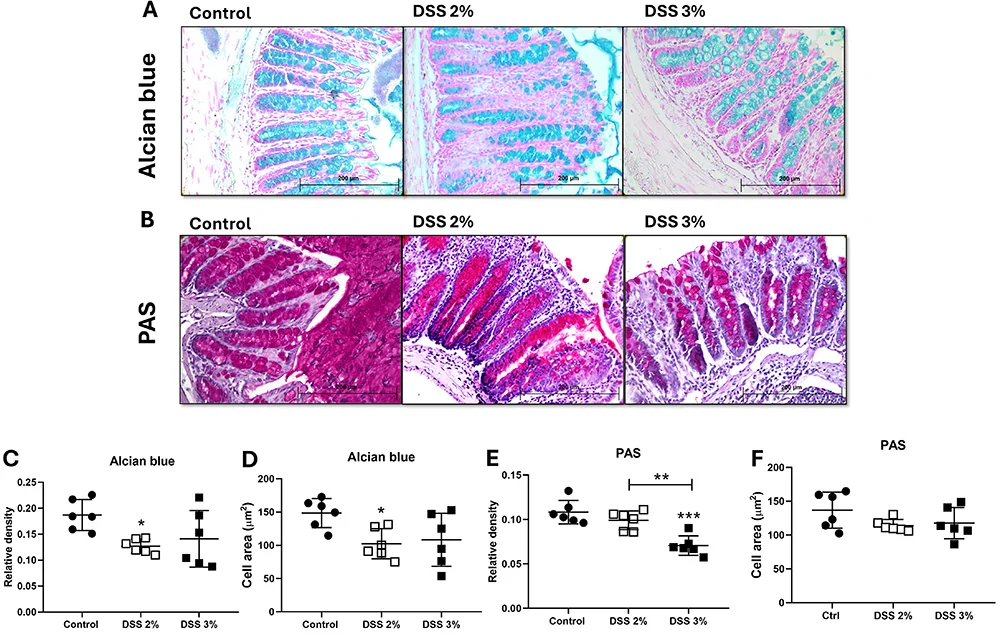
**A**, Alcian blue (AB) to detect acidic sugars and **B**, Periodic Acid-Schiff (PAS) stain for neutral sugars in colon samples of dextran sulfate sodium (DSS)-induced colitis of BALB/c mice treated with 2 and 3% DSS. **C**, Relative density of AB-positive cells per crypt length and **D**, AB positive cells per area (μm^2^). **E**, Relative density of PAS-positive cells per crypt length and **F**, PAS-positive cells per area (μm^2^). Data are reported as means±SD of 6 mice per group. *P<0.05, ***P<0.001 *vs* the control group; **P<0.01 between DSS-treated groups. ANOVA between DSS-treated groups. ANOVA and Holm-Sidak *post hoc* test.

AB staining, used as a marker of acidic sugars (Figure 4A), indicated that, compared to the control, the relative density (Figure 4C) and area (Figure 4D) of AB-positive cells were lower in the 2% DSS group (P<0.05). No other significant differences were found.

PAS staining, used as a marker of neutral sugars (Figure 4B), indicated that relative density in the 3% DSS group was lower compared to the control (P<0.001) and 2% DSS groups (P<0.01) (Figure 4E). No significant difference in cell area was observed (Figure 4F).

### Intestinal permeability was increased and mucopolysaccharide content was decreased by both DSS dosages

**Figure 5:**
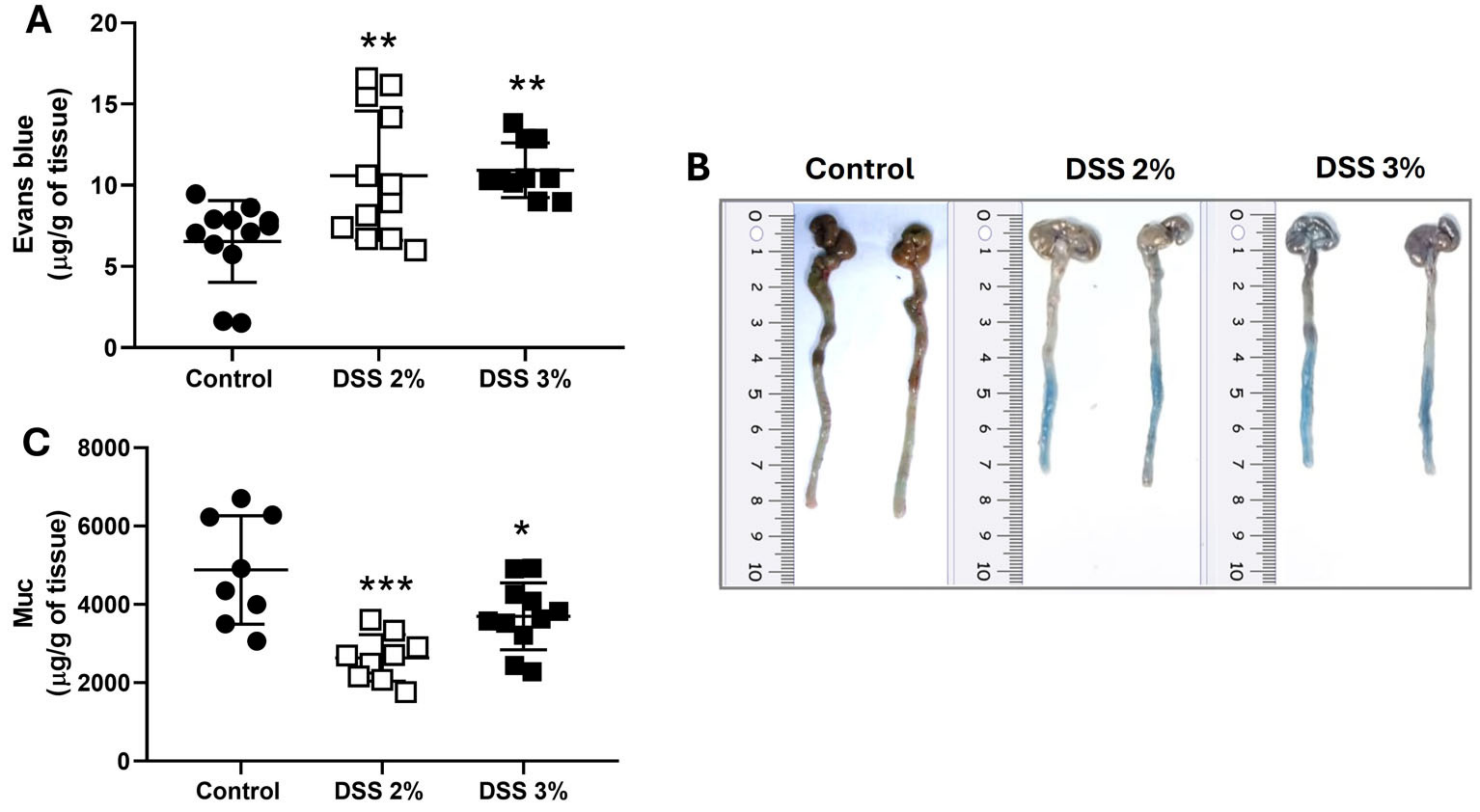
Intestinal permeability and mucopolysaccharide (Muc) content in colon samples of dextran sulfate sodium (DSS)-induced colitis BALB/c mice. **A**, Intestinal permeability computed as Evans blue concentration in intestinal tissue. **B**, Representative photographs of Evans blue permeation in colon samples of each experimental group. **C**, Muc concentration in colon tissue. Data are reported as means±SD of 8-12 mice per group. *P<0.05, **P<0.01, and ***P<0.001 *vs* the control group. ANOVA and the Holm-Sidak *post hoc* test.

Compared to the control group, intestinal permeability was significantly increased in both DSS-treated groups (P<0.01 for both comparisons), with no significant differences between the DSS-treated groups (Figure 5A and B). Mucopolysaccharide content was significantly lower in the 2% (P<0.001) and 3% (P≤0.05) DSS groups compared to the control (Figure 5C). No other significant differences were observed.

## Discussion

This study focused on optimizing the murine model of DSS-induced colitis in male BALB/c mice. Evaluating survival in the DSS-induced colitis model is crucial for establishing the optimal DSS dose for each mouse strain. Several studies in BALB/c mice have assessed survival following exposure to different DSS doses (10,17-20). Publishing results on this parameter provides guidelines for the ethical management of mice and supports the standardization of animal models.

According to the findings of this study, final survival was 80% with 2% DSS and 57% with 3% DSS. Therefore, 2% DSS was selected as the optimal dose for inducing colitis. In contrast, another study reported that administering 2% DSS by gavage once or twice daily for 7 consecutive days resulted in 100% survival, causing only colon length shortening without affecting body weight, DAI, histopathologic changes, or inflammatory markers (26). In other experimental settings with BALB/c mice, a 2.5% DSS dose caused no colitis symptoms (19), while other studies reported colitis symptoms with survival rates of 80 to 100% at DSS doses of 2.5% (10), 3.5% (17), and 5% (18,20).

The progression of DSS-induced murine colitis model is monitored by assessing DAI and/or body weight (16,22). In the current study, both DSS dosages increased DAI, with significant differences between the two doses observed on days 7, 9, and 14. Unlike these findings, in BALB/c mice administered 2% DSS for 10 days, no changes in DAI or body weight were observed (15). Previous experiments with BALB/c mice showed increased DAI with 3.5% DSS (17) or 5% DSS (20), while body weight loss and/or colon length shortening occurred with 2.5% DSS (16,22), 3.5% DSS (17), and 5% DSS (7,18).

In this study, colitis severity was also assessed by body weight. The 3% DSS dose caused both a decrease in body weight and 57% survival, whereas 2% DSS did not affect body weight and resulted in 80% survival.

Data suggested that DSS-associated body weight loss and mortality may be related to energy depletion and reduced food intake caused by inflammatory cytokines such as TNF-α, which promote protein catabolism (27). Colon length shortening was observed in both DSS-treated groups, with no significant differences between them. Colon length shortening is known to be associated with DSS-induced intestinal fibrosis due to increased collagen deposition (28).

HE staining of colonic samples from BALB/c mice showed inflammatory cell infiltration and colonic damage (7,10,13,16,17,20). In the current study, both DSS doses increased cellular infiltrate, with an apparent increase in hyperplasia and fibrosis in the 3% DSS group. Hyperplasia is a common feature of colitis, resulting from excessive epithelial repair and accumulation of transit-amplifying cells, leading to goblet cell depletion and thinning of the mucus layer (3).

Regarding goblet cell cellularity, 2% DSS reduced AB-positive cell density, with no significant differences between the two DSS doses. This finding aligns with reports of goblet cell depletion in DSS-induced colitis in BALB/c mice (10,29). Furthermore, both DSS doses decreased total mucopolysaccharide concentration, as measured by the AB test, consistent with the reduction of the major gel-forming mucin in the colon, mucin 2 (Muc2), as estimated by ELISA (29). Mucus depletion is known to allow bacterial penetration and contact with the epithelium, potentially leading to a proinflammatory response (5). The goblet cell and mucopolysaccharide depletion observed in this study may be related to dysbiosis-associated disturbances, which may result from the release of microbiota-derived secondary metabolites that modulate gene expression of products that regulate gut barrier integrity and function by reducing goblet cell count and luminal mucin-associated mucopolysaccharides (30). In this study, 3% DSS decreased the number of PAS-positive cells, markers of neutral sugars. The sugar profile is known to change under dysbiosis due to microbiota-derived glycosidases and, under inflammatory conditions, due to oxidative products that degrade glycans (31).

DSS-induced colitis increases intestinal permeability, which drives mucosal erosion and ulceration, as well as increased colonic infiltration of inflammatory cells such as polymorphonuclear leukocytes and macrophages in the lamina propria and submucosa (14). In the current study, DSS doses increased intestinal permeability as assessed by the Evans blue assay, consistent with findings in 5% DSS-treated BALB/c mice (14). In DSS-induced colitis, increased permeability has been associated with elevated translocation of luminal endotoxins into the bloodstream, leading to a heightened inflammatory response characterized by increased TNF-α levels (9,30).

The divergent results reported in this manuscript compared to other experiments using BALB/c mice may be related to several factors, including: i) the degree of sulfation, as higher DSS sulfation reduces cell viability (32); ii) divergent susceptibility to DSS of BALB/c mice from different vendors, which is determined in part by gut microbiota (33). To avoid disparities in behavior that affect differences in DSS consumption from drinking bottles, administration by gavage has been proposed to accurately control DSS dosage in mice (26); however, this approach induces a stress response due to handling.

A limitation of the present study was that the murine colitis model included only male BALB/c mice and did not include females. This is relevant because sex hormones, particularly estrogens, can modulate epithelial barrier integrity and immune responses in the gut (34). Therefore, future studies including female mice will be important to determine whether hormonal regulation influences the severity and reproducibility of the optimized DSS protocol.

Although DSS-induced colitis is one of the most widely used experimental models of ulcerative colitis, reproducibility remains a major challenge due to variations in DSS concentration, exposure time, and strain susceptibility. The present study provides a systematic comparison of two commonly used DSS concentrations (2 and 3%) in BALB/c mice, evaluating not only clinical outcomes of colitis severity but also key parameters related to intestinal barrier integrity, including permeability and mucopolysaccharide content. By integrating these endpoints with mortality and disease activity assessment, our results help define experimental conditions that balance effective disease induction with manageable mortality rates. This information is particularly relevant for laboratories studying mechanisms associated with epithelial barrier dysfunction, as excessive mortality or severe tissue damage may confound downstream analyses. Therefore, our findings support the use of an optimized 2% DSS concentration to achieve consistent colitis induction while preserving sufficient survival for mechanistic and therapeutic studies, improving the practical utility and reproducibility of this widely used model. Additionally, this study may provide insights into current knowledge of the mechanisms of colitis pathogenesis.

## Conclusions

In this study, long-term progression of acute colitis to evaluate potential relapses of DSS-induced colitis was not performed. Despite this, we concluded that under our working conditions, a 2% DSS concentration was optimal for inducing symptomatic colitis while maintaining high viability, adhering to ethical guidelines that prioritized minimizing animal suffering.

Improving the reproducibility and survival rate of animals is particularly relevant for mechanistic studies investigating inflammatory pathways and for preclinical evaluation of therapeutic strategies. By establishing experimental conditions that produce reliable clinical and histopathological outcomes, the optimized protocol described here may facilitate future studies aimed at understanding the pathogenesis of colitis and testing novel anti-inflammatory interventions.

## Data Availability

All data generated or analyzed during this study are included in this published article.
